# Group hypnotherapy versus group relaxation for smoking cessation: an RCT study protocol

**DOI:** 10.1186/1471-2458-12-271

**Published:** 2012-04-04

**Authors:** Maria Dickson-Spillmann, Thomas Kraemer, Kristina Rust, Michael Schaub

**Affiliations:** 1 Swiss Research Institute for Public Health and Addiction, Konradstrasse 32, 8031 Zurich, Switzerland; 2 Institute of Forensic Medicine, Zurich University, Winterthurerstrasse 190, 8057 Zurich, Switzerland

**Keywords:** Tobacco, Smoking cessation, Hypnosis, Relaxation, Cotinine, Randomised controlled trial

## Abstract

**Background:**

A significant number of smokers would like to stop smoking. Despite the demonstrated efficacy of pharmacological smoking cessation treatments, many smokers are unwilling to use them; however, they are inclined to try alternative methods. Hypnosis has a long-standing reputation in smoking cessation therapy, but its efficacy has not been scientifically proven. We designed this randomised controlled trial to evaluate the effects of group hypnosis as a method for smoking cessation, and we will compare the results of group hypnosis with group relaxation.

**Methods/Design:**

This is a randomised controlled trial (RCT) to compare the efficacy of a single session of hypnosis with that of relaxation performed in groups of 8-15 smokers. We intend to include at least 220 participants in our trial. The inclusion criteria include smoking at least 5 cigarettes per day, not using other cessation methods and being willing to quit smoking. The intervention is performed by a trained hypnotist/relaxation therapist. Both groups first receive 40 min of mental preparation that is based on motivational interviewing. Then, a state of deep relaxation is induced in the hypnosis condition, and superficial relaxation is induced in the control condition. Suggestions are made in the hypnosis condition that aim to switch the mental self-image of the participants from that of smokers to that of non-smokers. Each intervention lasts for 40 min. The participants also complete questionnaires that assess their smoking status and symptoms of depression and anxiety at baseline, 2 weeks and 6 months post-intervention. In addition, saliva samples are collected to assess cotinine levels at baseline and at 6 months post-intervention. We also assess nicotine withdrawal symptoms at 2 weeks post-intervention.

**Discussion:**

To the best of our knowledge, this RCT is the first to test the efficacy of group hypnosis versus group relaxation. Issues requiring discussion in the outcome paper include the lack of standardisation of hypnotic interventions in smoking cessation, the debriefing of the participants, the effects of group dynamics and the reasons for dropouts.

**Trial registration:**

Current Controlled Trials, ISRCTN72839675.

## Background

In 2010, 19% of the Swiss population between 14 and 65 years of age were daily smokers, and the average smoker smoked 14.2 cigarettes each day [1]. Although Switzerland ranks low in smoking prevalence in the European Union, it still ranks higher than the UK, Sweden, Portugal and the USA [2,3]. Smoking prevalence rates should be compared with caution, however, due to the different definitions of smoking and the various sampling methods [4]. Among daily and nondaily smokers in Switzerland, 26% have reported that they intend to quit smoking within the following 6 months [1]. In addition, other studies have reported a high willingness to quit smoking and high rates of cessation attempts [5-8].

The use of nicotine replacement therapy (NRT) has risen sharply in the past decade, and the efficacy of NRT delivered as gum, patches, nasal sprays, inhalers or tablets has been demonstrated [9,10]. Other pharmacological methods with proven efficacy in smoking cessation include the antidepressant bupropion and the nicotine receptor partial agonist varenicline [11]. The adverse effects of pharmacological cessation treatments are usually mild to moderate [11], which makes them generally safe to use.

Studies have also shown that smokers have concerns about pharmacological cessation methods, which makes them hesitant to use pharmacological methods. One study found that one-quarter of smokers worried about the possible side effects of NRT, and only 16% actually believed that NRT could help them quit smoking [12]. Smokers' worries about NRT might be influenced by inaccurate statistical comparisons between the success of NRT and the more commonly used "cold turkey" method. In addition, smokers tend to have a misperception of nicotine withdrawal symptoms as NRT side effects and false beliefs that nicotine is a major cause of tobacco-related health problems [13]. Nevertheless, these prior studies have indicated that not all smokers are willing to use pharmacological cessation methods to quit smoking. As a result, a vast spectrum of non-pharmacological methods has become available, ranging from self-help materials, counselling and advice on cognitive-behavioural therapy to methods of alternative medicine.

One form of alternative medicine that has been practiced for many years in smoking cessation is hypnosis [14]. On the basis of the American Psychological Association's Division 30 (Society of Psychological Hypnosis) definition, hypnosis describes the procedure "in which one person (the subject) is guided by another (the hypnotist) to respond to suggestions for changes in subjective experience, alterations in perception, sensation, emotion, thought, or behavior... Details of hypnotic procedures and suggestions will differ depending on the goals of the practitioner and the purposes of the clinical or research endeavour. Procedures traditionally involve suggestions to relax..." [15]. Different assumptions exist regarding the mode of action of hypnosis in smoking cessation. By acting on underlying impulses, hypnosis may weaken the desire to smoke, strengthen the will to stop or improve the ability to respond to a treatment programme. The success of hypnotherapy may also critically depend on factors such as the hypnotisability of subjects, nonspecific ceremonial, anticipatory and placebo factors or the relationship between the therapist and the subject [16].

Smokers are aware of hypnosis as a method of smoking cessation [17], and acceptance of this method among smokers seems high. One study reported that 67% of tobacco users expressed an interest in the future use of hypnosis for smoking cessation [18]. There is still insufficient scientific evidence, however, for the efficacy of hypnosis in smoking cessation, which is primarily due to the large variation in control interventions and missing information about hypnotic interventions. Systematic reviews repeatedly concluded that hypnotherapy has not been proved to have any greater effect on 6 month cessation rates compared with other interventions or no intervention at all [14,19]. These reviews recommended that large trials in which the type of hypnotherapy was clearly defined and described were needed to establish the efficacy of hypnosis in smoking cessation. In addition, comparisons should be made with active interventions of equal duration.

### Aims of the trial

The aim of the present trial is to investigate whether a single session of hypnosis evokes biologically validated higher rates of smoking abstinence than a single session of relaxation 2 weeks and 6 months following the intervention (primary outcome). Our trial is designed to comply with the recommendations that were presented in the systematic reviews of hypnotherapy in smoking cessation [14,19]. The hypnosis and relaxation sessions are performed in a single-session group format to evaluate them as economically feasible and time-efficient alternatives to existing methods of smoking cessation. To the best of our knowledge, this is the first randomised controlled trial (RCT) that investigates the effectiveness of group hypnosis. Secondary outcomes include nicotine withdrawal symptoms, smoking abstinence self-efficacy and symptoms of depression and anxiety. In this trial, we further aim to compare the cessation rates observed in our study with cessation rates following established and proven cessation treatments reported in other studies.

## Methods/Design

### Recruitment and randomisation

Recruitment is initiated through online and print advertisements. Upon first contact via e-mail or telephone, the prospective participants are asked to complete a form showing possible dates for the group hypnosis or relaxation (therapy) session. A therapy session is organised when 8 to 15 participants have signed up for the same date, and the participants are informed about the date and location of their session. Further study information is provided to the participants in advance, either in the context of a short informational meeting or through written materials. The participants are provided with the following information prior to the therapy session:

- No clear results exist regarding the efficacy of group hypnosis and relaxation in smoking cessation

- It is unclear which method is more successful

- Each participant has a 50% chance of being assigned to the hypnosis or relaxation condition

- Saliva samples will be taken at baseline and at 6 months post-intervention

The participants are assigned to the hypnosis or the relaxation group condition using an online randomisation program and remain unaware of their assignment until the end of the therapy session. The therapist is blind with regard to the respective condition until the conclusion of the first part of the intervention.

### Participants

Men and women are eligible to participate in the study if they smoke at least 5 cigarettes per day, are willing to quit smoking and are not currently using other methods of smoking cessation (see the overview in Table 1). The participants should understand and speak German, be between 18 and 65 years of age, not be intoxicated by alcohol or other substances (except for nicotine) before and during the intervention, not have a history of psychotic disorders and should not use stimulating medications (e.g., venlafaxine or methylphenidate). To ensure their commitment to smoking cessation and their motivation for the hypnosis or relaxation intervention, the participants contribute 40 Swiss Francs (*ca*. 37 USD) for their participation.

**Table 1:** Inclusion and exclusion criteria and reasoning

Inclusion Criteria	Reasoning
- Minimum age of 18 years, maximum age of 65	To ensure a minimum age of participation
- Smoking an average of at least 5 cigarettes per day	To ensure inclusion of regular smokers only
- Provision of informed consent	To ensure informed consent of the subjects
**Exclusion Criteria**	**Reasoning**
- Participation in other psychosocial or pharmacological interventions/therapies that could interfere with smoking cessation or any NRT treatments or medications for smoking cessation during the study	To avoid confounding treatment effects
- Acute alcohol or other substance use intoxication other than nicotine	To avoid confounding of alcohol or substance use effects
- Any signs of psychotic symptoms	To avoid subjects with these problems entering the study as symptoms could be exacerbated during relaxation or hypnosis

### Setting

**Figure 1:**
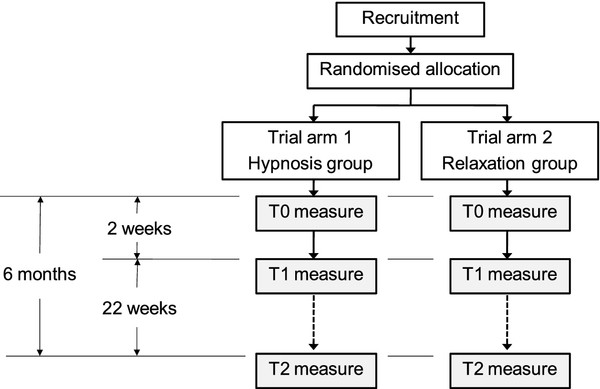
**Trial flowchart**. This figure provides an overview of the procedures for the participants.

The present study is undertaken by the Swiss Institute for Research in Public Health and Addictions (RIPA), which is associated with the University of Zurich. The therapy sessions take place in the conference rooms of hotels or the institutions involved, either in Zurich city or in a small town in Northwest Switzerland between 8-10 pm on weekdays and between 10-12 am on Saturdays. At the beginning of each session, the participants are welcomed by the project leader (first author), asked to turn off their mobile phones devices and instructed to complete the informed consent. The inclusion and exclusion criteria are provided in Table 1. Following detailed instructions, the participants are asked to use a saliva-measuring device and to complete a number of questionnaires. Then, the therapist is introduced to the participants, and the session begins. The therapist is a trained hypnotist and relaxation therapist with a private practice independent from RIPA. The follow-up assessments include a detailed telephone interview 2 weeks (t1) post-intervention by a trained scientific employee of RIPA and a postal assessment 6 months (t2) post-intervention (please see the trial flowchart in Figure 1).

### Measurement instruments

The primary outcome is the biological validation of a 30 day point prevalence of nicotine abstinence [20]. The following secondary outcome instruments are used to assess nicotine withdrawal symptoms, smoking abstinence self-efficacy and symptoms of depression and anxiety: the Minnesota Nicotine Withdrawal Scale (MNWS) [21]; the smoking abstinence self-efficacy assessment [22], short version translated into German [23]; the short version of the Beck Depression Inventory (BDI-V) [24], which is a derived, validated, and user-friendly German short version of the classical Beck Depression Inventory [25]; and the Beck Anxiety Inventory (BAI) [26], which was translated into German and validated [27].

At baseline, sociodemographic data about the participants (e.g., age, education, and nationality) and smoking-related variables (number and nature of previous cessation attempts, age at first cigarette, smoking relatives etc.) are collected. The following baseline instruments are also assessed: the Fagerström Test for Nicotine Dependence [28], which was translated into German and validated [29]; the "Fragebogen Substanzanamnese" (FDA), which ascertains the lifetime consumption, the past month's consumption and the manner of consumption for the DSM-IV/ICD-10 substances of abuse (this measure was derived from the EuropeASI) [30]; the Health Survey SF-12 [3], which was translated into German [31]; and the body mass index (BMI). A detailed overview of the intake and outcome assessments used during the course of the study is provided in Table 2.

**Table 2:** Measurements and instruments

Variable	Intake assessment (t0)	Two week follow-up (t1) - telephone interview	Six month follow-up (t2) - postal assessment
Sociodemographic information	Age, education, civil status		
Tobacco and other substance consumption	Fagerström Test for Nicotine Dependence FTND [28], German translation [29]; smoking abstinence self-efficacy [22], short version and German translation [23]; history of tobacco use and tobacco cessation attempts; history of substance use (FDA); Smoking friends and relatives; Age at first cigarette; Previous cessation attempts (number and nature); Body Mass Index	Minnesota Nicotine Withdrawal Scale MNWS [21]; smoking abstinence self-efficacy; point prevalence of tobacco abstinence (last 7 days)	Point prevalence of tobacco abstinence (last 30 days)
Mental health status	Beck Depression Inventory [25]; BDI-V, short version and German translation [24]; Beck Anxiety Inventory (BAI) [26], German translation [27]	BDI-V, BAI	BDI-V, BAI
General health status	SF-12 [3], German translation [31]	-	
Safety	-	Adverse events	Adverse events
Biological assessments	Salivary cotinine measurement	-	Salivary cotinine measurement
Misc. assessments	-	Use of CD	Use of CD

### Biological validation

To ensure validation of the primary outcome, biological validation is provided by salivary cotinine measurements. The concentration of cotinine, which is a metabolite of nicotine, is assed via liquid chromatography-mass spectrometry/mass spectrometry (LC-MS/MS) analysis at the Institute of Forensic Medicine of the University of Zurich.

#### Sample collection and cotinine determination

The saliva samples are collected using Quantisal^® ^saliva collection devices (nal von minden, Regensburg, Germany). A basic liquid-liquid extraction is employed using cotinine-d3 (c = 0.05 ng/μL) as an internal standard. The dried extracts are reconstituted in 100 μL of water containing ammonium formate. The sample is transferred to an autosampler vial, and 10 μL of sample is injected into the LC-MS/MS system. The analytes are separated using a Shimadzu integrated high-pressure liquid chromatography (HPLC) system followed by MS/MS detection using an AB Sciex 5500 Q Trap linear ion trap quadrupole mass spectrometer with Analyst software (AB Sciex, Darmstadt, Germany). Gradient elution is performed on a reversed-phase column (Synergi 4 μ POLAR-RP 80A, Phenomenex, Aschaffenburg, Germany). The mobile phase consists of 5 mM ammonium formate buffer, which is adjusted to pH 3.5 with formic acid (eluent A), and methanol containing ammonium formate (eluent B). The column oven is set at 40°C. Transitions for multiple-reaction monitoring (MRM) are selected, and their setting parameters are determined using Analyst Software in quantitative optimisation mode. The mass spectrometer is operated in the information-dependent acquisition mode. The MRM mode is used for the survey scan, and this is followed by the dependent scan, which is an enhanced product ion scan (EPI). Unambiguous identification is achieved by comparing the resulting EPI mass spectra with reference spectra from an in-house library. The three selected MRM transitions for cotinine are 177/80, 177/98 and 177/53, and the selected MRM transitions for cotinine-d3 are 180/80 and 180/53.

#### Quantitative analysis and cut-off

Calibration curves are prepared with cotinine-free saliva and buffer spiked with cotinine and IS in the following cotinine concentrations (ng/mL saliva): 1 ng/mL, 2 ng/mL, 4 ng/mL, 20 ng/mL, 40 ng/mL, 80 ng/mL, 400 ng/mL and 800 ng/mL. A calibration range of 1 to 80 ng/mL is used for low cotinine concentrations, whereas a range of 80 to 800 ng/mL is used for high concentrations. A spiked quality control containing 8 ng/mL cotinine is used in addition to an authentic sample with a known concentration of the analytes. To differentiate between active nicotine consumption and passive non-consumption, a cut-off value of 5 ng/mL saliva is used.

#### Handling of conflicting results and other problems

The participants whose saliva sample result is positive even though they state that they did not smoke during the follow-up period are asked for a second saliva sample. If the second saliva sample is also positive, then they will count as smokers in the intention-to-treat analysis. In the case that the first saliva sample is invalid or there is any suspicion of falsification, a second saliva sample is collected. If the second sample is still unclear, then the participant will count as a smoker.

The participants who provide informed consent for the study but refuse to provide a saliva sample will count as smokers. In addition, they are offered the option of having their partner or someone in close contact with them confirm their smoking status. Although the confirmations of close family members or friends have been shown to be reliable [32], the participants who do not provide a saliva sample will not be included in the primary outcome analyses (i.e., their results will be reported separately).

### Estimation of effect size

For the estimation of effect size, we refer to a study of Carmody et al. [33] that investigated the efficacy of hypnosis versus behavioural individualised counselling combined with nicotine patches. They recruited a total of 286 subjects, many of whom were war veterans. The interventions in the Carmody et al. study resulted in 30 day point prevalences of smoking abstinence of 26% in the intervention group and 18% in the control group. Although the Carmody et al. study conducted individual interventions, we aim to recruit motivated subjects and conduct group interventions. In the present study, we expect abstinence rates of approximately 30% in the hypnosis and 18% in the relaxation group condition. Aiming for a statistical power of 80% and accepting an alpha level of 5%, the target sample size for the current trial is 156 individuals. Assuming a dropout rate of 40%, we aim to include 220 participants.

### Interventions

The interventions are divided into three parts. The first part consists of psycho-education regarding smoking cessation and is based on the principles of motivational interviewing [34,35]. The participants' intentions to quit are reinforced through illustrative examples of the financial benefits of not smoking, smoking as a habit rather than an addiction, and the importance of attitude and commitment in smoking cessation. The participants are prepared for situations in their everyday life that will require enhanced attention and resistance, and they are educated about ways to deal with these situations. A flipchart is used to visualise important points. To reinforce change, the participants are designated by the therapist as non-smokers from the beginning of the session. After a short break during which the participants pay their contribution and the therapist is informed of the respective intervention, the second part of the session takes place with dimmed lights and soft background music.

The actual intervention occurs during the second part of the therapy session. In the hypnosis condition, a light hypnosis is induced through repetitive statements such as "you are going deeper and deeper into relaxation" or "you are very relaxed". The induction of hypnosis requires 4-5 min before the first set of suggestions is made to disconnect pleasant experiences, such as socialising or holidays, from the act of smoking. Hypnosis is then deepened by repeating statements involving relaxation and by associating ('*anchoring'*) the resulting state of deep relaxation with a key word that is subsequently repeated to maintain this state. During deep relaxation, the participants are given suggestions to switch their self-image from that of smokers to non-smokers. In addition, suggestions are made for the participants to use their power to resist smoking in tempting situations and to deal with symptoms such as mood swings or enhanced appetite, which may result as a consequence of smoking cessation [36-38]. At the end of the session, the participants are led back to full awareness.

In the relaxation condition, the participants are initially invited to make themselves comfortable and to relax. No repetitive statements are made, and no anchors are used to reinforce and deepen relaxation. The participants are asked to listen to the music for 4-5 min before the same suggestive sentences used in the hypnosis group are given; however, the participants in the relaxation group are not in a mental and physical state of hypnosis. The relaxation intervention lasts for the same amount of time as the hypnosis intervention (40 min).

In the third part of the intervention, the participants are debriefed about the study condition and given a CD for self-relaxation or self-hypnosis to repeat the potential effects of the intervention as frequently as they desire. On the CDs for each group, the trained hypnotist/relaxation therapist speaks to relaxing background music. In the self-hypnosis CD, the anchor is used to repeat the suggestions that are made during the group hypnosis. In the relaxation CD, corresponding sentences are repeated without anchoring and without being shifted to a mental and physical state of hypnosis. At the end of the session, any open questions are answered.

### Safety

During the 6 months duration of the study, the participants are offered the option of contacting the study team in case they experience any adverse events. In the introduction to the interventions, the participants are instructed to go to the nearest hospital and/or call an ambulance in case of unexpected emergencies. Moreover, adverse events are assessed systematically in the follow-ups at t1 and t2 to avoid under-reporting of adverse events (Figure 1).

### Data analysis

The data will be analysed according to the intention-to-treat principle. Intake measurements will be compared using t tests and chi-square tests. Differences between primary and secondary outcome variables at intake and 6 weeks will be tested using the generalised estimating equation (GEE) algorithms within the statistical package STATA 10 SE (StataCorp LP, College Station, Texas, USA). Effect sizes and risk ratios will be calculated for primary and secondary outcomes where appropriate. Explorative predictor and moderator analyses on primary outcomes will be performed according to Kraemer et al. [39]. In addition, we will conduct exploratory regression analyses to test whether intake variables predict nicotine abstinence, smoking abstinence self-efficacy and nicotine withdrawal (MNWS). For these analyses, we will use linear, multinomial, or binary regression models depending on the scale level of the outcome measures.

### Handling of study dropouts

Subjects that withdraw their informed consent or are not available for the follow-up assessments at t1 and t2 will count as dropouts. Reasons for dropping out in the participants who withdraw from informed consent are assessed as soon as possible by a telephone interview and will be reported systematically in the study dissemination process.

### Ethical review

This RCT is executed in compliance with the Declaration of Helsinki and has been reviewed by the Ethics Committee of the Canton of Zurich, which did not declare any objections (KEK-StV-Nr.16/10).

## Discussion

To the best of our knowledge, this RCT is the first study to test the efficacy of group hypnosis versus group relaxation. It is also be the first study to explore the efficacy of group hypnosis to reduce nicotine withdrawal symptoms, symptoms of depression and anxiety that potentially occur during smoking cessation and whether smoking abstinence self-efficacy can be increased by hypnosis. Moreover, predictor and moderator analyses of primary outcomes will be performed.

There are some potential limitations regarding the present study that will merit discussion in the main report. First, we attempted to design our study in a manner that would allow it to be comparable to other studies of hypnotherapy and smoking cessation. For example, we followed the recommendations of Barnes et al. and Abbot et al. [14,19] when designing the study; however, no standardised protocol exists for hypnotic interventions in smoking cessation. There are some elements that are common to most interventions, such as those named by Spiegel (e.g., that a body is entitled to protection from smoke) [40], but there are many degrees of freedom in the implementation of hypnotic procedures that may affect the outcomes. The definition of hypnosis suggested by APA Division 30 confirms the relatively large scope regarding the induction and execution of hypnosis. Furthermore, it is plausible that the nonspecific factors mentioned by Spiegel [16] may influence the outcome of hypnotic intervention, and such nonspecific factors complicate comparisons between studies. To approach the problem of non-standardisation, we will provide more details on the nonspecific factors and on the precise execution of the interventions (e.g., the wording of suggestions) in our main study report.

Another potential concern is that the participants are told about their study condition during the debriefing, and some may even realise it by themselves during the intervention. In the case that a participant prefers one condition to the other, they might be disappointed, and this could lower their motivation to resist smoking after the session. Various considerations, however, led us to the decision to reveal the study conditions to the participants. We want to prevent any uncontrollable effects of speculations, insecurities or convictions by the participants regarding their study condition from mixing with the effects of the interventions on the outcome variables. Moreover, our intent is to evaluate group hypnosis and relaxation as regular treatments to be offered to the public and integrated into the healthcare system, and patients would be aware of their treatment in the real-world implementation of hypnosis and relaxation as smoking cessation treatments. This point actually makes our disclosure a strength of the study.

Group interventions might be influenced by group dynamics, which could potentially affect a participant's experience of the intervention and its outcome. Single individuals, for example, may dominate the group through sarcastic remarks or other behaviours. We are taking qualitative records of such group dynamics as they occur, and we plan to include them in our data interpretation as much as possible. The fact that the participants may have attended alone or in the company of an acquaintance also represents a variable in our data collection system.

After the therapy session, we advise our study participants not to use any other smoking cessation methods during the next 6 months to avoid confounding factors of other interventions. As we have no possible way to control the participants' behaviours during this period, we have to rely on self-reports about their use of other smoking cessation therapies. Throughout the recruitment, the therapy sessions and the follow-ups, however, we emphasise to our participants that their honest information is crucial to our study results and to the current state of knowledge in science. In addition, we tell them that there will not be any consequences if they report trying out other methods. We hope that by conveying this message to our participants, they are motivated to cooperate throughout the study and provide us with honest reports about their experiences after their therapy session.

## Competing interests

The authors declare that they have no competing interests. This trial is registered with Current Controlled Trials and is traceable as ISRCTN72839675.

## Authors' contributions

MDS prepared the first draft of the paper and coordinated the study. MS is the principle investigator, developed the study design and prepared the final draft of the paper. TK critically reviewed the study design and developed the methods for analysing saliva. KR coordinated and analysed the saliva samples. All of the authors approved the final version of the manuscript that was submitted for publication.

## Pre-publication history

The pre-publication history for this paper can be accessed here:

http://www.biomedcentral.com/1471-2458/12/271/prepub

## References

[B1] Keller R Radtke T Krebs H Hornung R Der Tabakkonsum der Schweizer Wohnbevölkerung in den Jahren 2001 bis 2010. Tabakmonitoring - Schweizerische Umfrage zum Tabakkonsum 2011 Zurich: Psychologisches Institut der Universität Zurich, Sozial- und Gesundheitspsychologie

[B2] Bogdanovica I Godfrey F McNeill A Britton J Smoking prevalence in the European Union: a comparison of national and transnational prevalence survey methods and results Tob Control 2011 20 e4 10.1136/tc.2010.036103 20966129 PMC3003865

[B3] Ware JE Kosinski M Turner-Bowker DM Gandek B How to score version 2 of the SF-12 health survey (with a supplement documenting version 1) 2002 Lincoln: QualityMetric Incorporated

[B4] Bogdanovica I Jiang GX Löhr C Schmidtke A Mittendorfer-Rutz E Changes in rates, methods and characteristics of suicide attempters over a 15-year period: comparison between Stockholm, Sweden, and Wurzburg, Germany Soc Psychiatry Psychiatr Epidemiol 2011 46 1103 1114 10.1007/s00127-010-0282-3 20820754

[B5] Etter JF Perneger TV Ronchi A Distributions of smokers by stage: international comparison and association with smoking prevalence Prev Med 1997 26 580 585 10.1006/pmed.1997.0179 9245682

[B6] McCaul KD Hockemeyer JR Johnson RJ Zetocha K Quinlan K Glasgow RE Motivation to quit using cigarettes: a review Addict Behav 2006 31 42 56 10.1016/j.addbeh.2005.04.004 15916861

[B7] Tobacco Use Supplement to the Current Population Survey (TUS-CPS) http://riskfactor.cancer.gov/studies/tus-cps/results/data0607/table3.html

[B8] Aubin HJ Peiffer G Stoebner-Delbarre A Vicaut E Jeanpetit Y Solesse A Bonnelye G Thomas D The French Observational Cohort of Usual Smokers (FOCUS) cohort: French smokers perceptions and attitudes towards smoking cessation BMC Public Health 2010 10 100 10.1186/1471-2458-10-100 20184784 PMC2841669

[B9] Stead LF Perera R Bullen C Mant D Lancaster T Nicotine replacement therapy for smoking cessation Cochrane Database Syst Rev 2008 1 CD000146 DOI: 10.1002/14651858.CD000146.pub3 18253970 10.1002/14651858.CD000146.pub3

[B10] Moore D Aveyard P Connock M Wang D Fry-Smith A Barton P Effectiveness and safety of nicotine replacement therapy assisted reduction to stop smoking: systematic review and meta-analysis BMJ 2009 338 b1024 10.1136/bmj.b1024 19342408 PMC2664870

[B11] Lancaster T Stead L Cahill K An update on therapeutics for tobacco dependence Expert Opin Pharmacother 2008 9 15 22 10.1517/14656566.9.1.15 18076335

[B12] Etter JF Perneger TV Attitudes toward nicotine replacement therapy in smokers and ex-smokers in the general public Clin Pharmacol Ther 2001 69 175 183 10.1067/mcp.2001.113722 11240982

[B13] Mooney ME Leventhal AM Hatsukami DK Attitudes and knowledge about nicotine and nicotine replacement therapy Nicotine Tob Res 2006 8 435 446 10.1080/14622200600670397 16801301

[B14] Barnes J Dong CY McRobbie H Walker N Mehta M Stead LF Hypnotherapy for smoking cessation Cochrane Database Syst Rev 2010 10 CD001008 DOI: 10.1002/14651858.CD001008.pub2 20927723 10.1002/14651858.CD001008.pub2

[B15] Green JP Barabasz AF Barrett D Montgomery GH Forging ahead: the 2003 APA Division 30 definition of hypnosis Int J Clin Exp Hypn 2005 53 259 264 10.1080/00207140590961321 16076663

[B16] Spiegel D Frischholz EJ Fleiss JL Spiegel H Predictors of smoking abstinence following a single-session restructuring intervention with self-hypnosis Am J Psychiatry 1993 150 1090 1097 8317582 10.1176/ajp.150.7.1090

[B17] Hammond D McDonald PW Fong GT Borland R Do smokers know how to quit? Knowledge and perceived effectiveness of cessation assistance as predictors of cessation behaviour Addiction 2004 99 1042 1048 10.1111/j.1360-0443.2004.00754.x 15265101

[B18] Sood A Ebbert JO Sood R Stevens SR Complementary treatments for tobacco cessation: a survey Nicotine Tob Res 2006 8 767 771 10.1080/14622200601004109 17132524

[B19] Abbot NC Stead LF White AR Barnes J Hypnotherapy for smoking cessation Cochrane Database of Systematic Reviews 1998 2 CD001008 DOI:10.1002/14651858.CD001008 10.1002/14651858.CD001008 10796583

[B20] Hughes JR Keely JP Niaura RS Ossip-Klein DJ Richmond RL Swan GE Measures of abstinence in clinical trials: issues and recommendations Nicotine Tob Res 2003 5 13 25 12745503

[B21] Hughes JR Hatsukami D Signs and symptoms of tobacco withdrawal Arch Gen Psychiatry 1986 43 289 294 10.1001/archpsyc.1986.01800030107013 3954551

[B22] DiClemente CC Prochaska JO Gibertini M Self-efficacy and the stages of self-change of smoking Cognit Ther Res 1985 9 181 200 10.1007/BF01204849

[B23] Jaekle C Keller S Baum E Basler HD Skalen zur Selbstwirksamkeit und Entscheidungsbalance im Prozeß der Verhaltensänderung von Rauchern Diagnostica 1999 45 138 146 10.1026//0012-1924.45.3.138

[B24] Schmitt M Maes J Vorschlag zur Vereinfachung des Beck-Depressions-Inventars (BDI) Diagnostica 2000 46 38 46 10.1026//0012-1924.46.1.38

[B25] Beck AT Steer RA Beck Depression Inventory (BDI) 1987 San Antonio: The Psychological Corporation Inc

[B26] Beck AT Epstein N Brown G Steer RA An inventory for measuring clinical anxiety: psychometric properties J Consult Clin Psychol 1988 56 893 897 3204199 10.1037//0022-006x.56.6.893

[B27] Margraf J Ehlers A Beck-angst-inventar (BAI). Deutschsprachige adaptation des Beck anxiety inventory 1995 Huber: Bern

[B28] Heatherton TF Kozlowski LT Frecker RC Fagerström KO The Fagerstrom Test for Nicotine Dependence: a revision of the Fagerstrom Tolerance Questionnaire Br J Addict 1991 86 1119 1127 10.1111/j.1360-0443.1991.tb01879.x 1932883

[B29] Bleich S Havemann-Reinecke U Kornhuber J Der Fagerström-Test für Nikotinabhängigkeit (FTNA) 2002 Göttingen: Hogrefe

[B30] Kokkevi A Hartgers C Europe ASI: European adaptation of a multidimensional assessment instrument for drug and alcohol dependence Eur Addict Res 1995 1 208 210 10.1159/000259089

[B31] Bullinger M Kirchberger I Ware JE Der deutsche SF-36 Health Survey. Übersetzung und psychometrische Testung eines krankheitsübergreifenden Instrumentes zur Erfassung der gesundheitsbezogenen Lebensqualität Zeitschrift für Gesundheitswissenschaften 1995 1 21 3619349661

[B32] Chen Y Rennie DC Dosman JA The reliability of cigarette consumption reports by spousal proxies Am J Public Health 1995 85 1711 1712 10.2105/AJPH.85.12.1711 7503354 PMC1615718

[B33] Carmody TP Duncan C Simon JA Solkowitz S Huggins J Lee S Delucchi K Hypnosis for smoking cessation: a randomized trial Nicotine Tob Res 2008 10 811 818 10.1080/14622200802023833 18569754

[B34] Miller WR Rollnick S Motivational interviewing: preparing people for change 2002 New York, London: Guilford Press

[B35] Tober G Raistrick D Motivational dialogue: Preparing addiction professionals for motivational interviewing practice 2007 London, New York: Routledge

[B36] Lynn SJ Neufeld V Rhue JW Mattorin A Rhue JW, Lynn SJ, Kirsch I Hypnosis and smoking cessation: A cognitive-behavioral treatment Handbook of clinical hypnosis 1993 Washington DC: American Psychological Association 555 585

[B37] Green JP Linn SJ, Kirsch I, Rhue JW Cognitive-behavioral hypnotherapy for smoking cessation: a case study in a group setting Casebook of clinical hypnosis 1996 Washington DC: American Psychological Association 223 248

[B38] Green JP Kirsch I, Capafons A, Cardena-Buehn E, Amigó S Hypnosis and the treatment of smoking cessation and weight loss Clinical hypnosis and self-regulation: Cognitive behavioral perspectives 1999 Washington DC: American Psychological Association 249 276

[B39] Kraemer HC Wilson GT Fairburn CG Agras WS Mediators and moderators of treatment effects in randomized clinical trials Arch Gen Psychiatry 2002 59 877 883 10.1001/archpsyc.59.10.877 12365874

[B40] Spiegel H A single-treatment method to stop smoking using ancillary self-hypnosis Int J Clin Exp Hypnosis 1970 18 235 250 10.1080/00207147008415923 5457608

